# Designing and describing an electronic referral system to facilitate direct hospital admissions

**DOI:** 10.1186/s12875-022-01656-4

**Published:** 2022-03-28

**Authors:** Marion Montellier, Raphaëlle Delpech, Mathieu Mion, François Boué, Marie-Hélène Metzger

**Affiliations:** 1 University of Paris-Saclay, Department of General Practice, 94276 Le Kremlin-Bicêtre, France; 2 University of Paris-Saclay, UVSQ, INSERM, CESP, 94807 Villejuif, France; 3 Assistance Publique - Hôpitaux de Paris, Antoine-Béclère Hospital, Geriatric Service, Clamart, France; 4 Assistance Publique - Hôpitaux de Paris, Antoine-Béclère Hospital, Internal Medicine Service, Clamart, France; 5 OSMOSE Health Network, Clamart, France

**Keywords:** Continuity of patient care, Primary health care, General practitioners, Medical staff, Hospital, Health information exchange, Admitting Department, Hospital, Medical informatics applications

## Abstract

**Background:**

In France, the progressive use of emergency departments (EDs) by primary care providers (PCPs) as a point of access to hospitalization for nonurgent patients is one of the many causes of their overcrowding. To increase the proportion of direct hospital admissions, it is necessary to improve coordination between PCPs and hospital specialists. The objective of our work was to describe the design and implementation of an electronic referral system aimed at facilitating direct hospital admissions.

**Methods:**

This initiative was conducted in a French area (Hauts-de-Seine Sud) through a partnership between the Antoine-Béclère University Hospital, the Paris-Saclay University Department of General Medicine and the local health care network. The implementation was carried out in 3 stages, namely, conducting a survey of PCPs in the territory about their communication methods with the hospital, designing and implementing a web-based application called “SIPILINK” (Système d'Information de la Plateforme d’Intermédiation Link) and an innovative organization for hospital management of the requests, and analysing through descriptive statistics the platform use 9 months after launch.

**Results:**

The e-referral platform was launched in November 2019. First, a PCP filled out an electronic form describing the reason for his or her request. Then, a hospital specialist worked to respond within 72 h. Nine months after the launch, 132 PCPs had registered for the SIPILINK platform, which represented 36.6% of PCPs in this area. Of the 124 requests made, 46.8% corresponded to a hospitalization request (conventional or day hospitalization). The most requested specialty was internal medicine (48.4% of requests). The median time to first response was 43 min, and 43.5% of these requests resulted in direct admission (conventional or day hospitalization).

**Conclusions:**

This type of system responds to a need for coordination in the primary-secondary care direction, which is less often addressed than in the secondary-primary care direction. The first results show the potential of the system to facilitate direct admissions within a short time frame. To make the system sustainable, the next step is to extend its use to other hospitals in the territory.

**Supplementary Information:**

The online version contains supplementary material available at 10.1186/s12875-022-01656-4.

## Background

In France, hospital emergency rooms have been in crisis for many years. The model conceived in the 1960s was centred on the management of life-threatening emergencies and serious traumas. However, these activities have changed substantially over the years, and emergency rooms have become a source of chronic overcrowding [1]. The same observation has been made at the international level [2, 3]. Part of this overcrowding is related to inappropriate emergency department (ED) visits. The lack of standardization of this concept makes the estimates of published rates not very comparable [4]. It has been estimated that between 13.5% and 27.4% of the ED visits that occurred in 2013 in France were ‘nonurgent’ [5] and that 16.1% of visits in England between 2015–2017 fell under the same category [6]. Many studies have been conducted to identify the causes of these inappropriate ED visits. Most of these studies have focused on patient characteristics (social vulnerability, need for free care, anxiety, consumerist attitude, etc.) [7] or on the inadequacy of the primary care offer (lack of preventive activities, medical deserts) [4, 7, 8].

The reorganization of the system is becoming a public health priority. One area for improvement is to facilitate direct admissions, which are defined as hospital admissions that do not go through the ED [9]. However, this direct admission process requires good coordination between primary care providers (PCPs) and hospital specialists to ensure the fluidity of the primary-secondary care pathway. Few studies have addressed the impact of the lack of primary-secondary care coordination on the progressive use of the ED by PCPs as a gateway to hospitalization for nonurgent patients [9].

In France, Derame et al. showed that 10% of patients hospitalized via an ED visit could have benefited from direct admission [10]. The authors concluded their study by remarking on the need to set up telephone hotline systems between PCPs and hospital specialists to facilitate direct admission. The investigation conducted by this team in 2008 showed that despite the implementation of such a system, direct admission was still not scheduled often enough. More than one patient out of three was still hospitalized in downstream wards via an ED visit during working hours even though they were eligible for direct admission [11]. Dijon et al. experimented with a telephone hotline for the direct admission of geriatric patients, which showed a reduction in the length of hospitalization in the group that benefitted from direct admission and a reduction in the time to rehospitalization [12]. However, the use of a telephone hotline poses a certain number of problems described by Hermant, namely, difficulty in reaching the hospital specialist, time spent scheduling hospitalization and the occasional loss of information about the request, which results in the absence of progress in the management of the patient [13].

The use of digital tools can help overcome these difficulties. Various electronic referral systems have been tested in North America and have been shown to allow general practitioners to make nonurgent requests to specialists [14]. These systems have been proven to be effective by reducing the time needed to access specialists and reducing the costs associated with hospitalizations [14, 15]. These systems have thus enabled quantitative and qualitative improvements in communication between PCPs and hospital doctors. However, they were designed for requests for specialist advice or consultations but not for direct hospital admission.

The objective of our study was to describe the development and implementation of an electronic referral system in an area of the Paris suburbs (Hauts-de-Seine Sud—France) aiming to facilitate direct hospital admissions.

## Methods

### Experiment location

This experiment was carried out in the Hauts-de-Seine Sud (92-South area) coordination area located in the Paris suburbs (France). This area is home to approximately 600,000 inhabitants [16]. The experiment was conducted through a partnership among the Antoine-Béclère University Hospital, the Paris-Saclay University Department of General Medicine and the DAC-OSMOSE health network (see Additional file 1: focus).

### Survey of PCPs on their communication methods with the Antoine-Béclère Hospital

To gather information about the communication methods that existed prior to the implementation of our system, we conducted a survey of PCPs in the Hauts-de-Seine Sud 92 area using a self-administered questionnaire. We were able to contact 302 out of the 361 PCPs listed in the area. The questionnaire was distributed to these individuals from March 2019 to October 2019, either by delivering it during in-person meetings or by dropping the questionnaire off at their medical practice.

### Development and implementation of the primary-secondary care coordination system: the “intermediation platform”

Due to a high rate of inappropriate ED visits (5% during a pilot study conducted in 2017), staff from Antoine-Béclère Hospital wished to initiate brainstorming on the subject. A working group was created that consisted of representatives from management (*n* = 2) and hospital downstream wards (1 in internal medicine, 1 in geriatrics) and the heads of the ED (*n* = 1) and the Assistance Publique—Hôpitaux de Paris (AP-HP) information technology department (*n* = 2). The work outcome was the development of a new system with the objective of coordinating medical decisions between hospital doctors and PCPs and programming the corresponding hospital care. This system was called the "intermediation platform". It was established that the hospital’s response to a PCP request should be made within 72 h. This 72-h time frame was established by consensus within the working group and was supported by the literature review (i.e., the same 72-h time frame is found in Kim et al. [17]).

It appeared essential that this new system be coupled with the development of a digital tool that facilitated communication between PCPs and hospital doctors. Two representatives of the University Department of General Medicine of Paris-Saclay as well as two hospital medical secretaries joined the working group.

A first prototype of the digital tool called SIPILINK (Système Information de la Plateforme d'Intermédiation Link) was developed and tested by the different user panels of the working group.

The primary-secondary care coordination system was launched in November 2019. The PCPs installed in the study area were informed of its implementation via a face-to-face meeting organized in March 2019 by the 3 partners of the project (AP-HP, DAC-Osmose, DUMG). Several mailings were sent out to complete the information provided to PCPs.

### Analysis of the use of the primary-secondary care coordination system 9 months after the launch

A descriptive analysis of the system’s use was carried out 9 months after its launch to study the dynamics of its implementation. This analysis was carried out using data extracted from the SIPILINK information system.

As seen in the study by Tuot et al. [18], we used two effectiveness indicators, namely, the time taken to respond to the request and the time taken to access a hospital resource (consultation, conventional or day hospitalization).

## Ethics approval and consent to participate

The methods were performed in accordance with the relevant French guidelines and regulations. The survey of PCPs was declared to our hospital Data Protection Officer (DPO) and recorded in the data protection register of Paris-Saclay University Hospital. The medical data were collected as part of the SIPILINK patient medical management. Consequently, this study did not fall within the framework of French regulations on research involving human persons, and written patient consent was not required. A patient’s nonobjection was collected by the PCP (an informational note detailed the purpose and processing of the data) before the data were entered. As the data were processed by the hospital staff managing this care, this study did not require additional authorisation from an IRB or from the Ethical and Scientific Committee for Research, Studies and Evaluations in the field of Health (paragraph 2 of article 65 of French law no. 78–17 of 6 January 1978: https://www.cnil.fr/fr/la-loi-informatique-et-libertes#article65).

## Results

### Methods of communication between PCPs and Antoine-Béclère Hospital

Fifty-six of the 302 physicians to whom the questionnaire was distributed responded to the questionnaire, for a response rate of 18.5% (see Table 1). The survey found that 98.2% of the physicians questioned had contact with Antoine-Béclère Hospital (*n* = 55). Physicians contacted the hospital for medical advice (80.0%), medical emergencies (74.5%), outpatient consultations (63.6%), conventional hospitalization (61.8%) and, slightly less frequently, for day hospitalization (50.9%) (the sum of the percentages is higher than 100% because it was possible to choose several answers). The medical specialties most involved in these reciprocal relationships were internal medicine, hepato-gastro-enterology and gynaecology.

**Table 1:** Quality of communication between primary care practitioners (PCPs) and Antoine-Béclère Hospital (results of the survey conducted in Hauts-de-Seine Sud (France) between March and October 2019)

Item	N	%
	(*n* = 55)	
**Overall quality of communication with the hospital practitioner**		
Poor or weak	6	10.9
Moderate	8	14.5
Good or excellent	40	72.7
**Ease of getting in touch with the hospital practitioner**		
Poor or weak	20	36.3
Moderate	28	50.9
Good or excellent	6	10.9
**Ease of obtaining medical advice**		
Poor or weak	19	34.5
Moderate	23	41.8
Good or excellent	10	18.2
**Ease of obtaining an outpatient consultation**		
Poor or weak	23	41.8
Moderate	25	45.5
Good or excellent	4	7.3
**Ease of obtaining hospitalization**		
Poor or weak	16	29.0
Moderate	25	45.5
Good or excellent	7	12.7
**Patients referred to the ED**^**a**^** because the PCP could not reach the hospital**		
Never	8	14.5
Rarely	13	23.6
Sometimes or often	32	58.2

^*a*^*ED* Emergency department

The most common methods of communication were telephone calls for 81.8% of the physicians surveyed and e-mail for 32.7% of them. For 52.8% of the physicians surveyed, the confidentiality of the communication channels was moderate or even insufficient.

While 72.7% of the physicians felt that they had either good or excellent communication with hospital practitioners, only 10.9% of them rated the ease of getting in touch with her or him as either good or excellent. In total, 18.2%, 7.3%, and 12.7% of respondents rated the ease of obtaining medical advice, outpatient consultation or hospitalization as either good or excellent, respectively.

Thus, 18.2% of the PCPs declared that they often referred patients to the ED because they could not reach a hospital practitioner, and 40.0% reported that they sometimes did so.

### Description of the primary-secondary care coordination system

**Fig. 1:**
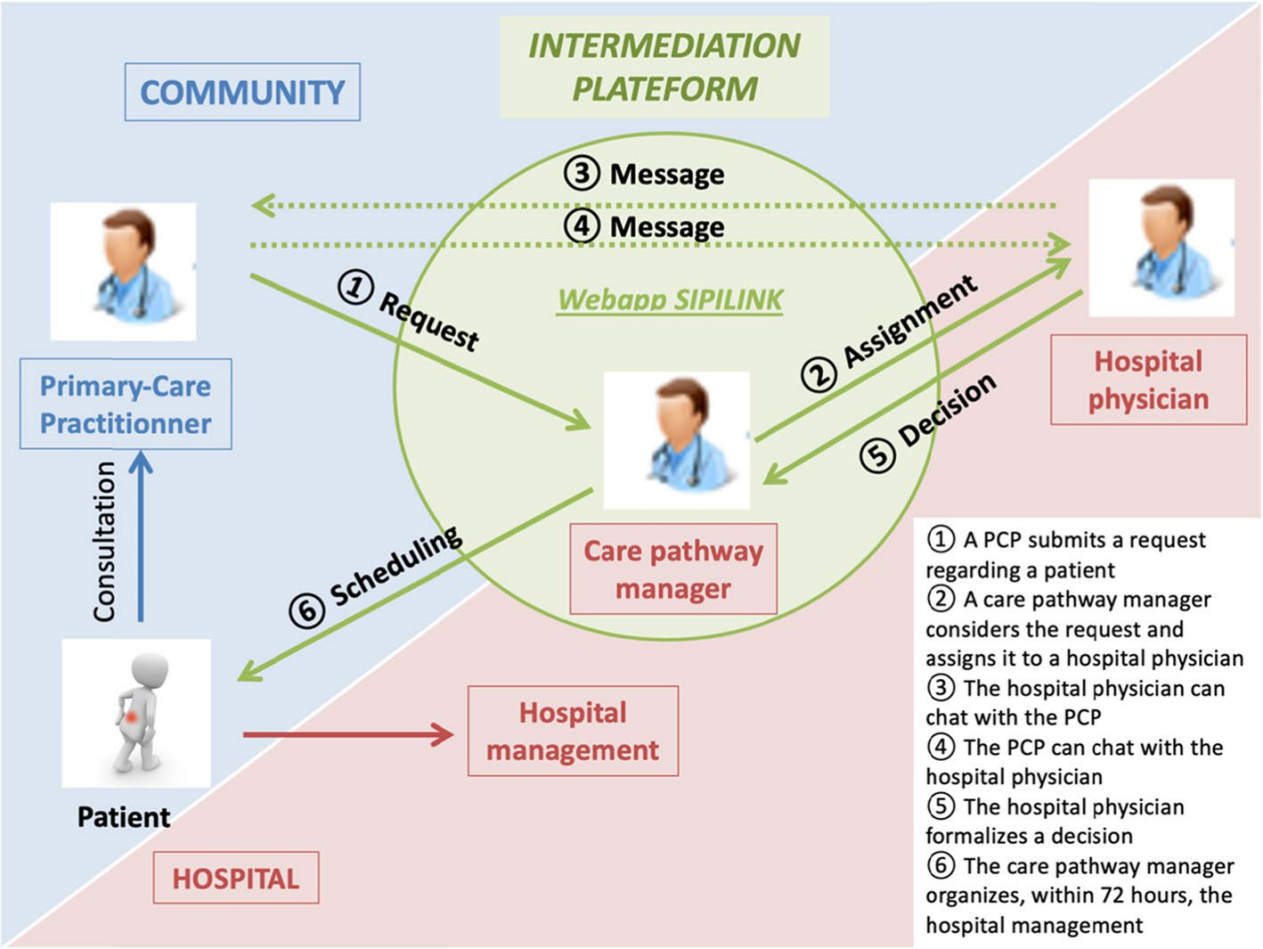
Coordinated care decision and programming process

The purpose of the intermediation platform is to organize a coordinated process of decision-making and to schedule the corresponding hospital care (see Fig. 1).

#### On the PCP’s side

The PCP who wishes to exchange information with a hospital physician for one of his or her patients (excluding true emergencies) uses SIPILINK, which is a web-based application that is accessible via a secure URL link. The application is accessible on any computer with internet access (PC, tablet, smartphone). The PCP must first register by creating an account and agreeing to the user terms. He or she then fills out an e-form to collect the administrative and medical information about the patient concerned. He or she then specifies the type of care he or she is considering for the patient (conventional hospitalization, day hospitalization, outpatient consultation) and whether he or she is requesting simple advice see supplementary material, additional file 2: screen shots of the e-form. The PCP can attach documents to facilitate medical decisions (photos, biological tests, prescriptions for medication). He or she can then choose the hospital medical specialty and the expressed response time (an “as soon as possible response time” or response time ranging from 24 to 72 h). Next, he or she can specify the medical reason for the request in 2 steps, namely, by selecting a reason from a pre-established list and by describing the medical situation and the query in free text form in a dedicated box. The list of reasons was pre-established based on the literature review [19–23] and from the clinical experiences of the physicians in the working group (see Table 2). This list was adapted to each medical specialty participating in the experimentation. If no reason is found that matches the request, the PCP can choose "other" and describe the reason in the box in free text form.

**Table 2:** Pre-established list of medical reasons in the SIPILINK e-form for the geriatric specialty

Specialty	Reason wording
Geriatrics	Recurrent falls
	Confusion
	Loss of autonomy
	Deteriorated state of health
	Abnormal weight loss (> 10%)
	Asthenia
	Unexplained fever (> 38.2 °C for more than 7 days) Unexplained anaemia (Haemoglobin < 8 g/dL)
	Other haematological abnormality of the blood count or splenomegaly or gammopathy Persistent unexplained adenopathy Arthralgias or other diffuse bone pain
	Other reason

When the request is processed by the hospital (care pathway manager and/or hospital physician), the PCP receives an alert by e-mail with the URL link of the request, which allows him or her to access the e-form and view the message sent and/or the medical decision made by the hospital physician.

#### On the hospital side

The participation of hospital wards in the intermediation platform is voluntary. To date, the participating wards include adult medical wards, namely, internal medicine (infectiology, diabetology, oncology, immunology, downstream services from ED), geriatrics, haematology, hepato-gastroenterology, gynaecology, addictology for pregnant women and prenatal diagnosis.

The hospital referral request made by the PCP is managed at the hospital level by the care pathway manager and the hospital doctor. The total number of hospital staff registered on the platform was 82 as of July 2020.

##### *Care pathway manager*

The task of care pathway managers is to coordinate interactions between PCPs and hospital doctors and to plan care pathways. The term "care pathway manager" was chosen because the position refers to different hospital job profiles depending on the type of hospital pathway to be planned, i.e., medical secretary for outpatient consultations, hospital secretary for conventional hospitalization or coordinating nurse for day hospitalization. These care pathway managers are positioned within the participating departments.

To carry out their tasks, care pathway managers have the ability to view the dashboard of requests made for the service to which they are authorized see supplementary material, Additional file 3: screen shots of the dashboard. They receive an alert by e-mail as soon as progress in the processing of the request is traced in the application.

In addition, a new job profile, namely, "cross-disciplinary care pathway managers", was created. Their mission is to ensure that requests are handled by the services solicited and, if necessary, to redirect requests to a medical specialty other than the one initially selected by the PCP. They also oversee the request processing in terms of care pathway scheduling to ensure that all requests from PCPs are processed through to the end of the scheduling process. A public health physician and a medical secretary currently perform this role at Antoine-Béclère Hospital.

##### *Hospital physician*

The management of the request is also based on the medical organization of each hospital department. A "hotline" must be set up within the department to enable a rapid and appropriate response within the time limits mentioned by the PCP. The SIPILINK digital tool ensures the continuity of responses in the case of the leave or absence of one of the team's doctors, as requests are accessible to all doctors within the specialty requested.

In case there is a request for an "as soon as possible” response time from the PCP in the e-form, the hospital doctor on duty via the "hotline" is informed by SMS. The SMS is received on a dedicated smartphone owned by the "hotline" doctor, which allows him or her to access the request directly (URL link inserted in the SMS). If the response time is between 24 and 72 h, the care pathway manager chooses the hospital physician who seems most appropriate to respond. The hospital physician is then notified by e-mail. He or she can also redirect the request to one of his or her colleagues at any time. Once the hospital physician agrees to process the request, he or she can respond to the PCP either by e-mail or by telephone to the PCP to discuss the case.

When a medical decision is made, the hospital physician traces it on SIPILINK and enters the information needed to program the care pathway, which is then processed by the relevant care pathway manager. This traceability enables information to be shared within the hospital team and with the PCP see supplementary material, Additional file 4: screen copies of medical decisions. When the programming has been completed, the care pathway manager closes the request, which is then archived on the hospital server that hosts the application.

### Analysis of the system’s use 9 months after launch

**Fig. 2:**
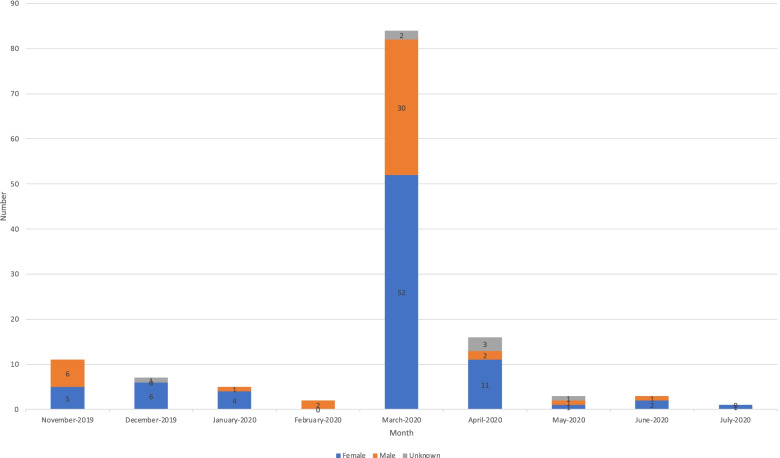
Distribution of primary care physicians enrolled over the first 9 months of use by month and gender (November 2019-July 2020)

After 9 months of implementation, 132 PCPs were registered on SIPILINK (i.e., 36.6% of the PCPs in the study area). After a slow start to registration, a registration peak occurred in March and April 2020, which corresponded to the first epidemic peak of SARS-CoV-2 infection in France. During these 2 months, 100 registrations were completed, which represented 75.6% of all registrations (see Fig. 2). Female physicians represented 62.1% of the registered PCPs.

One hundred and twenty-four requests were made during these first 9 months. Alongside the registration peak, there was a request peak (69 requests, i.e., 55.6% of total requests) during the period of March–April 2020.

### Request characteristics

The average age of patients for whom a request was made was 61.2 years (SD: 23.6) for women and 65.2 years (SD: 19.3) for men. The majority of the requests concerned patients who lived in cities near Antoine-Béclère Hospital.

Of the 124 requests, 46.8% concerned a request for hospitalization (29.8% for conventional hospitalization; 16.9% for day hospitalization), and 53.2% concerned a request for advice or consultation.

**Fig. 3:**
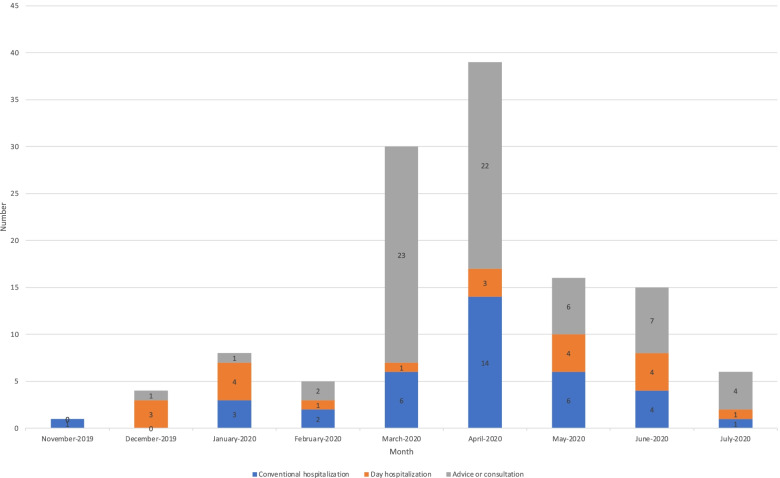
Distribution of SIPILINK requests by type of management requested by the primary care provider (November 2019-July 2020)

The proportion of requests for advice was greatly increased during the SARS-CoV-2 epidemic peak in the spring, with requests for advice constituting 76.7% and 56.4% of the requests made in March and April 2020, respectively (see Fig. 3). If we exclude these 2 months from the analysis, we observe that of the 55 requests, 61.8% concerned a request for hospitalization (30.9% for conventional hospitalization, 30.9% for day hospitalization) and 38.2% concerned a request for advice.

The most requested specialty was internal medicine (48.4%), followed by requests for COVID-19 management (21.0%), geriatrics (15.3%) and haematology (8.1%). When requests related to COVID-19 were excluded, internal medicine became predominant, constituting 61.2% of the requests. The average number of requests per month was 6.7 for internal medicine and 2.1 for geriatrics over this period.

The response time requested by the PCP was most often "within 72 h" (37.1%), followed by "within 24 h" (33.9%). The response time “as soon as possible" was chosen in only 17.7% of the requests.

COVID-19 represented 30.6% of the medical reasons for making contact during this period (16.1% for hospital care and 14.5% for advice). The most frequent reasons aside from COVID-19-related reasons were a biological abnormality (18.5%) and deteriorated state of health or alteration of general condition (8.9%) (See Table 3).

**Table 3:** Medical reasons for SIPILINK requests over the first 9 months of use (November 2019-July 2020)

Medical reason	n	%
Biological abnormality	23	18.5
COVID-19 hospital care	20	16.1
COVID-19 advice	18	14.5
Deteriorated state of health	11	8.9
Hepato-gastroenterology	7	5.6
Recurrent falls	6	4.8
Therapeutic advice	6	4.8
Cardiology	4	3.2
Immunology	4	3.2
Confusion – loss of autonomy	4	3.2
Advice for biological interpretation	4	3.2
Gynaecology	3	2.4
Infectiology	3	2.4
Oncology	2	1.6
Diabetology	2	1.6
Endocrinology	2	1.6
Advice for antenatal diagnosis	2	1.6
Nephrology	1	0.8
Neurology	1	0.8
Otorhinolaryngology	1	0.8
Total	124	100.0

### Hospital response characteristics

The median hospital response time to the request was 43 min (min = 1 min; 1st quartile = 18 min; 3rd quartile = 292 min or 4 h and 52 min; max = 6600 h or 4 days and 14 h). The hospital response was issued within the first hour for 59.7% of the requests, and only 10.5% of the requests had a response time of more than 24 h.

Patient management consisted of hospitalization for 43.5% of the requests (conventional hospitalization: 30.6%; day hospitalization: 12.9%). The average hospitalization time was 5.8 days (standard deviation: 9.6 days), and the median was 2 days. For 37.1% of the requests, only advice was given without hospital management. For 18.5% of the requests, the management consisted of a consultation. The average delay for a consultation appointment was 8.2 days (standard deviation: 9.9), and the median was 5 days.

## Discussion

A primary-secondary care coordination system to facilitate direct admission was implemented with the aim of increasing the rate of direct admissions to the hospital. The system was based on a digital tool, namely, SIPILINK, which was designed to address coordination difficulties. Communication difficulties were one of the causes reported in our survey of PCPs within the study area.

The participation rate for the PCP survey was low (18.5%). Low participation of general practitioners in surveys is a problem frequently described in the literature [24]. Explanatory factors with material and immaterial influences have been studied, and the participation rate has been shown to have important variability due to the context of each country, varying between 6% in Germany and Austria and 90% in Malta (median participation rate: 30%) [24]. We did not find any article in the scientific literature specifically studying the participation rate of general practitioners in declarative surveys in France, but by consulting several thesis works on equivalent subjects, we found various participation rates ranging from 5% [25] to 37% in the DEMOMED 75 study [26]. There is no current consensus on a method to be adopted to improve these participation rates. It seems that electronic survey methods often have lower response rates than paper mailed surveys [27]. Therefore, we favoured face-to-face contact to propose the survey. Despite this methodological limitation, the results obtained on our sample were in line with results published in the literature and reinforced our interest in developing an innovative coordination system. Indeed, the PCPs stated in our survey that they were satisfied with their collaboration with hospital physicians, particularly with the quality of their exchanges. This is consistent with the results of Marshall et al. [24]. However, the survey found that the frequent use of referral to the ED as a point of entry to hospitalization was due to the difficulty of contacting a hospital practitioner to schedule a direct admission.

One of the pillars of the system that was put in place was to allow a system of exchanges that optimized the medical time on both the hospital and primary care sides. On the primary care side, the interest was in avoiding the waiting time on the phone for PCPs to access a specialist who is able to respond to their request. On the hospital side, the interest in the system was in preventing specialists from interrupting their hospital activities with telephone calls, thus risking answering too quickly without having all the elements necessary to make the best decision. Indeed, the system allows the hospital to access the requests when they are available to respond to them under good conditions, possibly after accessing the patient's hospital medical file to specify the response and to start the care planning. Finally, the system allows for the traceability of exchanges as well as the sharing of these exchanges among all of the actors involved in the care (PCPs, hospital physicians, care pathway managers).

The description of the response times expressed by the PCPs showed that requests for a response "as soon as possible" were not frequent (17.7%); rather, the most frequently requested timeframe was within 72 h (37.1%), followed by a timeframe within 24 h (33.9%). The results concerning the real response times carried out by the hospital showed that the system largely met the doctors' expectations in terms of response time since only 10.5% of these responses were made after 24 h. This responsiveness is an extremely important point for the project’s sustainability. This result was possible due to the implementation of cross-disciplinary care pathway managers who monitored and reminded the hospital physicians in cases of nonresponse. In e-referral schemes evaluated by Tuot et al., 91–95% of the responses were made within 72 h, and the median time for an electronic response from a specialist was 24 h in the Los Angeles Safety Net Program eConsult System [28].

The SIPILINK system was particularly useful during the first peak of the SARS-CoV-2 pandemic in France, with a massive enrolment of PCPs in March and April 2020. The choice of technology (web-based application) as well as the development carried out by the hospital's IT team allowed for a high level of responsiveness when the pandemic started. It was indeed possible to create a new section almost instantaneously to address the needs related to COVID-19. This organizational innovation thus enabled PCPs to feel less isolated during this period of health crisis and to facilitate access to specialized advice and hospital care during a particularly tense period in terms of access to hospital care. Moreover, the satisfaction of PCPs with this type of system has been found in the international literature [29].

The development of this system also presented certain difficulties. On the hospital side, the compartmentalization of project management between hospital administration and doctors made it difficult to provide on-the-ground projects and did not facilitate the implementation of this project [30]. Thus, the first few months of implementation were relatively slow due to obstacles at both the hospital and primary care levels. Hospital staff are currently overworked and lack the time and availability to participate in innovations [31]. PCPs are sometimes entrenched in their practice habits and reluctant to change. These observations are not specific to the French health care system; similar observations have been noted internationally. In a review of the literature, Osman et al. described the most commonly mentioned obstacles to the use of e-consultation systems by general practitioners, namely, the need to modify their behaviour, the modification of their workload, and the loss of immediate contact with their usual specialist referrers or, in contrast, contact with specialists with whom they are not used to collaborating [29]. Our system differs in its purpose from the electronic addressing systems developed in the USA and Canada [18] because these systems are essentially aimed at referral to hospital specialist consultations, whereas our system is essentially aimed at referral for direct hospital admission. The impact of interventions on direct admission and their potential effects on reducing the rate of inappropriate emergency room visits has rarely been studied in the literature.

Moreover, few studies have examined the impact of the lack of coordination between the PCP and the hospital physician on inappropriate emergency room visits or on the rate of direct admission [9], thereby showing that this dimension, which we seek to address with our system, has not yet been the subject of much research. Andronikof et al. showed in a study published in 2008 that it was possible to organize scheduled hospitalization in only 40% of cases where hospitalization was indicated. In 11% of the cases, the solution proposed by the hospital contact person was to refer the patient to the emergency room [32]. As Leyenaar et al. pointed out, direct admission to the hospital is not suitable for all clinical situations [33]. It is indeed more appropriate for patients who require urgent care or a rapid imaging examination [9]. This is why we have excluded the management of urgent situations in the SIPILINK users’ agreement. The urgent nature of care is thus left to the discretion of the PCP. The reasons for requests in our study showed that the reasons were very rarely related to an emergency evaluation, with the exception of requests related to COVID-19. A before-and-after analysis of the direct admission rate after the implementation of the system could not be carried out because of interference with the SARS-CoV-2 health crisis. Indeed, the care pathway for admitted patients was modified in spring 2020, with systematic passage to the ED for admitted patients to test them for COVID-19 diagnosis. COVID-19 patients at the height of the spring 2020 epidemic wave constituted almost all of the patients who were hospitalized in the study hospital; furthermore, since the epidemic is still ongoing, this parameter is currently not assessable.

The difficulties of primary-secondary care coordination are most often addressed in the literature in the hospital primary care context [9, 34, 35] and essentially from the communication aspect, particularly in relation to hospital discharge. For example, Ekwegh et al. developed a model discharge letter (structured letter) to improve this communication [34]. In the primary care hospital context, delays in access to a specialist consultation are most often studied, with delays estimated at between 6 and 12 months. The estimated consequences are the loss of chance linked to a delayed diagnosis, the repetition of diagnostic examinations, the cost to the health system [36], and inappropriate visits to EDs [28]. Various e-addressing systems for specialist consultations have been tested in the USA and Canada [14, 17, 18, 37, 38] and more recently in England [39].

The first experimentation found in the literature was conducted at San Francisco General Hospital in 2005 [38]. A web portal was developed by this hospital to set up a primary-secondary care e-referral system. The system is very similar to the one we set up with the SIPILINK system. Each department has a designed medical referent who reviews and responds to requests from PCPs. He or she can then quickly schedule a consultation or hospitalization or ask for additional information or investigations before deciding on the patient's referral. These exchanges are then integrated into the hospital's electronic medical record [40]. This process is relatively comparable to that implemented with SIPILINK. In Canada, Liddy et al. described the development of a platform called "The Champlain BASE" (Building Access to Specialist through E-consultation), which is an application that allows primary care physicians to submit a request for nonurgent specialty care [14]. This platform was set up in 2010, and more than 10,000 requests were submitted within the first 5 years of operation. The authors were able to show that the platform reduced the time required to schedule a specialist consultation and, in particular, that 3.4% of the requests for advice for which the PCP did not initially consider a consultation resulted in a consultation. Similarly, a platform called e-Consult was developed in 2012 in the Los Angeles area to facilitate access to specialist consultations by filtering requests from PCPs via an e-form [28]. The e-form is then read by a doctor of the requested specialty who decides, according to the elements provided, whether to refer the patient to a consultation for the specialty requested. This system has seen a rapid increase in the number of requests made by PCPs and has reduced the time it takes to gain access to specialist doctors. The median response time is 24 h, and 25% of e-consultations are resolved without a physical consultation.

The reason for the lack of work on the impact of primary-secondary care coordination on the rate of direct admission is probably linked to the fact that most of the work on primary-secondary coordination is carried out by PCPs who have pointed out the consequence of this lack of coordination on primary care management, particularly after hospital discharge. Few hospital doctors have taken an interest in primary-secondary care coordination to analyse the difficulties generated on the hospital side. In summary, it seems that the subject of primary-secondary coordination has not yet been sufficiently decompartmentalized between the two health care sectors to improve the bottlenecks in the entire process [41].

The sustainability of the project is based on 3 factors: communication, territorial approach, and incentives. The start-up of the system was indeed slow but was boosted by the COVID-19 crisis. As the first wave of COVID-19 started a few weeks after the launch of the system, we were only able to do very little communication. In the context of a health crisis during which professionals are overwhelmed with information, we therefore favoured word of mouth among professionals. This process was relatively effective. Nine months after the launch, without any particular communication, 36.6% of the PCPs installed in the area were registered. To maintain the link with users and bring new PCPs on board, we will send a biannual newsletter to all PCPs in our territory, with the objective of reporting on the use and evolution of the service. This electronic distribution will be ensured by the general practitioner trainers’ network of the Paris Saclay University and by the Osmose territorial health network, whose role is to promote local initiatives by health professionals.

Another lever of sustainability is to extend the use of this primary-secondary care coordination system to other hospitals in the territory. It is indeed important that PCPs do not have to use different types of electronic communication tools depending on the hospital to which they wish to refer a patient. Currently, one of the challenges physicians face is the increasing number of digital solutions that are aimed at addressing certain sequences of the care pathway. This fragmented multiplication of tools will require knowledge about and the manipulation of numerous interfaces that may hinder the proper management of the patient’s care pathway. A model such as that developed in San Francisco can be adapted to the organization of our local health system [38]. The integration of the system into the shared electronic medical record currently under construction [42] will be one of the elements that determines its generalization. The third lever of sustainability of the project will also require the consideration of the remuneration of the actors involved in this coordination. Indeed, as doctors emphasized in a study in Catalonia, organizational factors such as coordination time are just as important as digital tools [43], which means that funding must be provided. Finally, Ekwegh et al. insisted on the importance of collaborative work between PCPs and hospital doctors for the development of their tool, a collaborative tool that is quite similar to the tool that we have developed in our project [34]; however, there are still very few formalized primary-secondary care networks that aim to develop collaboration and perpetuate this type of initiative [44]. Thus, it is necessary to develop them.

## Conclusion

The initiative presented in this publication has been supported by local stakeholders, which is a key element of its success. The first months of its implementation have shown its value in reducing the isolation of PCPs in their practice (particularly during the first wave of COVID-19) and in better organizing hospital responses to primary care requests to encourage direct admissions. To ensure its sustainability, the next step will be to extend its use to other hospitals in the region, which, in the current context of an abundance of digital offerings for PCPs, is the next challenge of the project. The deployment of digital tools for primary-secondary care coordination must be done within the framework of a territorial strategy for the health care organization defined by local actors and supported by the health authorities.

## Supplementary Information

**Additional file 1.**

## Data Availability

Survey data from PCPs about their communication methods and system use data 9 months after launch are available from the corresponding author upon reasonable request.

## Abbreviations

ED: Emergency department
PCP: Primary-care provider
SIPILINK: Système d'Information de la Plateforme d’Intermédiation Link
