# Supplementary material for: Designing and describing an electronic referral system to facilitate direct hospital admissions
Source: BMC Prim Care. 2022 Mar 28;23:57. doi: 10.1186/s12875-022-01656-4 (PMC8958479; doi:10.1186/s12875-022-01656-4)
Supplement: Supplementary file 1 — Additional file 1. [file 12875_2022_1656_MOESM1_ESM.pdf]

Additional files

Additional file 1: Focus on the coordination territory of Hauts-de-Seine Sud (Ile-de-France, France)

The Hauts-de-Seine Sud corresponds to an administrative coordination territory (92-Sud) defined by the regional authorities. This territory is composed of the following 15 municipalities: Antony, Clamart, Bagneux, Bourg la reine, Chatenay-Malabry, Chatillon, Fontenay aux Roses, Issy les Moulineaux, Le Plessis Robinson, Malakoff, Meudon, Meudon la Forêt, Montrouge, Sceau, Vanves.

URL link: https://www.ors-idf.org/fileadmin/DataStorageKit/ORS/Etudes/2019/territoireCoordination/92_sud_2019.pdf

Antoine-Béclère Hospital

Antoine-Béclère Hospital is a hospital of the Assistance Publique - Hôpitaux de Paris (AP-HP) and is the only public medical-surgical-obstetrical establishment in its territory. Other details are as follows:

- It is located in the town of Clamart in the Hauts-de-Seine Sud region;
- It has 1 adult and 1 paediatric emergency department, 6 hospitalization departments, 1 neonatal intensive care unit and 1 adult intensive care unit, and 1 ambulatory medicine and surgery centre;
- It has 389 hospital beds (including 11 beds for short-term hospitalizations < 24 h);
- It had 64,040 emergency room visits in 2018;
- It had 23,297 hospitalizations in 2018; and
- It boasts expertise in medically assisted reproduction and bariatric surgery.

URL link: http://hopital-antoine-beclere.aphp.fr

The University Department of General Medicine Paris-Saclay (DUMG)

The main missions of the DUMG are as follows:

- The training of general medicine residents;
- Participation in the teaching of 2nd cycle medical students and the organization of their internships in general medicine;
- The development of research projects related to primary care; and
- Participation in the life of the faculty.

URL link: https://www.medecine.universite-paris-saclay.fr/la-faculte/services-administratifs/departement-de-medecine-generale

The DAC-OSMOSE coordination support structure

DACs are unique legal entities financed by regional health agencies. They are staffed by a multiprofessional team (doctors, nurses, social workers) to organize the support of health professionals in the care pathways of patients in complex situations. This support is provided at the following two levels:

1. Individual support for the coordination of care paths:
   - o Information and referral to resources in the territory.
   - o Support for the organization of complex health care pathways (overall assessment of the person's needs; the development and proposal of a personalized assistance program; the mobilization, coordination and follow-up of the assistance plan; and the fluidity and coordination of communication between the stakeholders accompanying the patient).
2. Territorial support for the organization of care paths:

o Observation of care pathways to analyse the prevention of risks of breakdown.

o Coordination of operational cooperation between territorial partners.

URL link: http://www.reseau-osmose.fr

Additional file 2: Screenshots of the French SIPILINK e-form for requests from primary care physicians

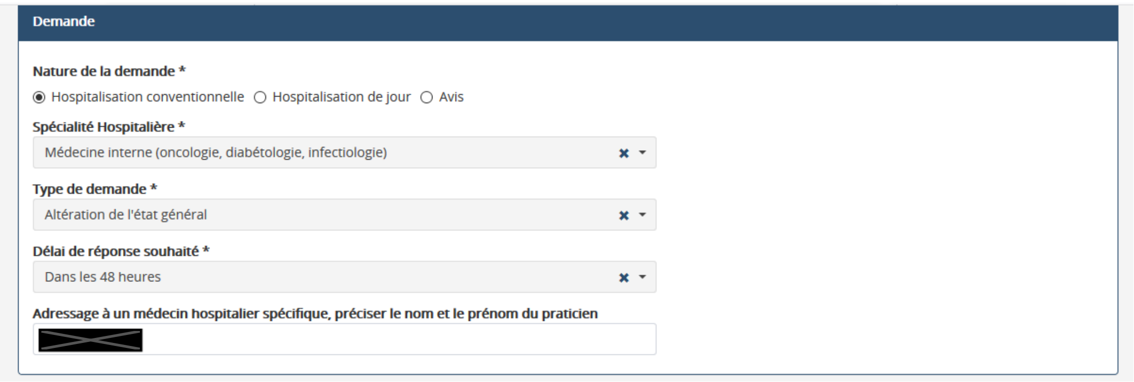

Demande

Nature de la demande *

(•) Hospitalisation conventionnelle ( ) Hospitalisation de jour ( ) Avis

Spécialité Hospitalière *

Médecine interne (oncologie, diabétologie, infectiologie)

Type de demande *

Altération de l'état général

Délai de réponse souhaité *

Dans les 48 heures

Adressage à un médecin hospitalier spécifique, préciser le nom et le prénom du praticien

[redacted]

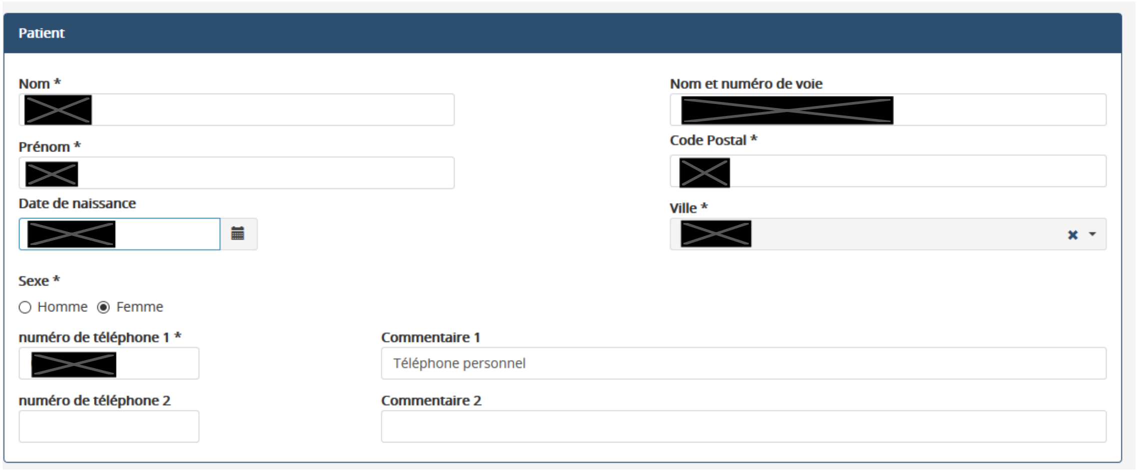

Patient

Nom *

[redacted]

Prénom *

[redacted]

Date de naissance

[redacted]

Nom et numéro de voie

[redacted]

Code Postal *

[redacted]

Ville *

[redacted]

Sexe *

( ) Homme (•) Femme

numéro de téléphone 1 *

[redacted]

Commentaire 1

Téléphone personnel

numéro de téléphone 2

Commentaire 2

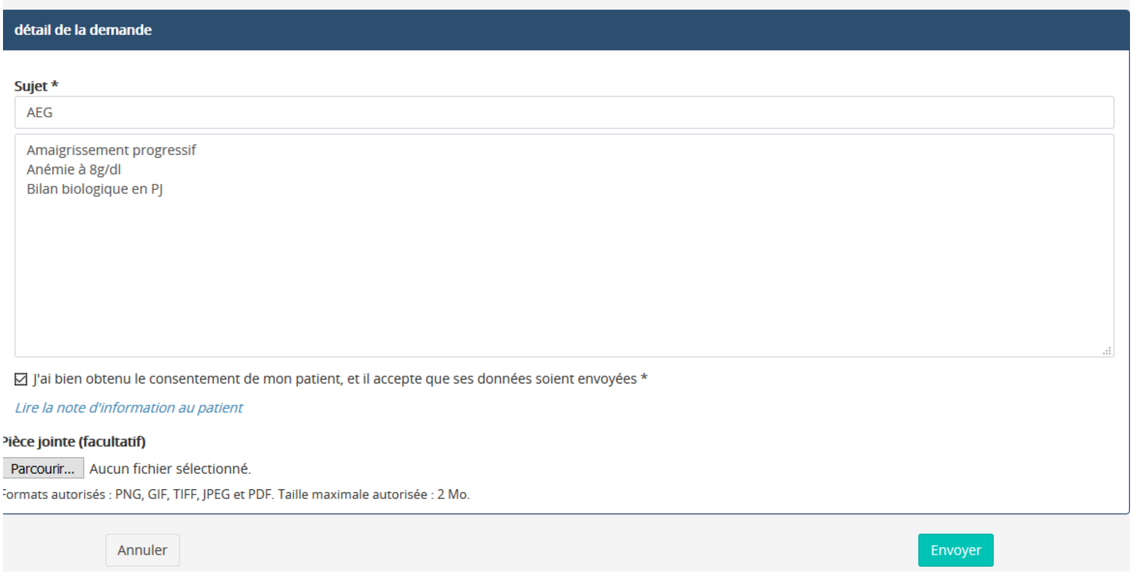

détail de la demande

Sujet *

AEG

Amaigrissement progressif
Anémie à 8g/dl
Bilan biologique en PJ

☑ J'ai bien obtenu le consentement de mon patient, et il accepte que ses données soient envoyées *

Lire la note d'information au patient

Pièce jointe (facultatif)

Parcourir... Aucun fichier sélectionné.

Formats autorisés : PNG, GIF, TIFF, JPEG et PDF. Taille maximale autorisée : 2 Mo.

Annuler Envoyer

Additional file 3: Screenshot of the French SIPILINK hospital dashboard

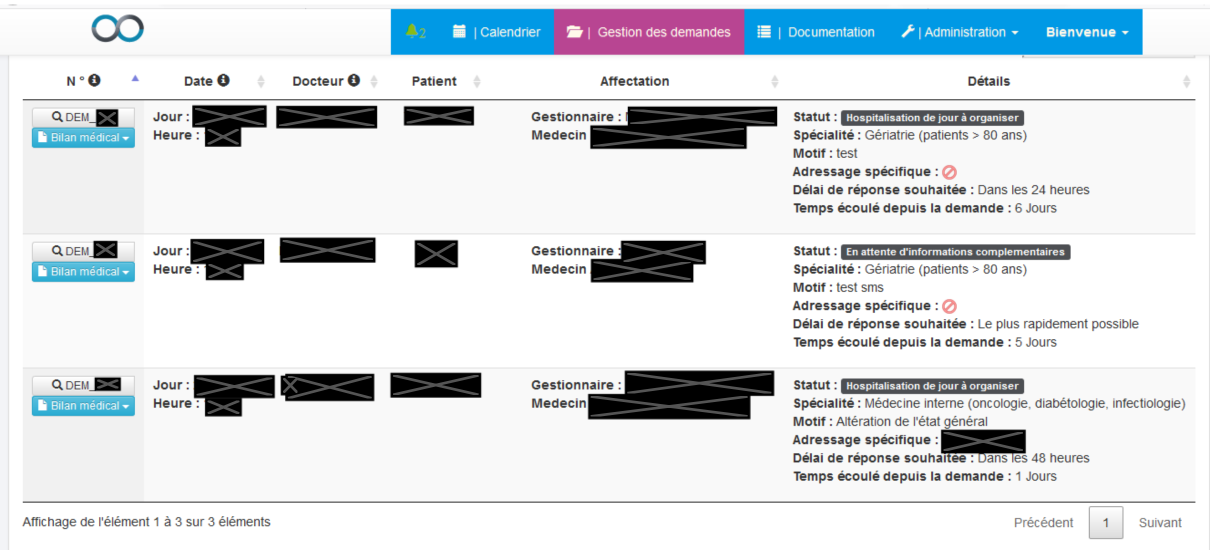

Additional file 4: Screenshots of the French SIPILINK hospital decision e-form

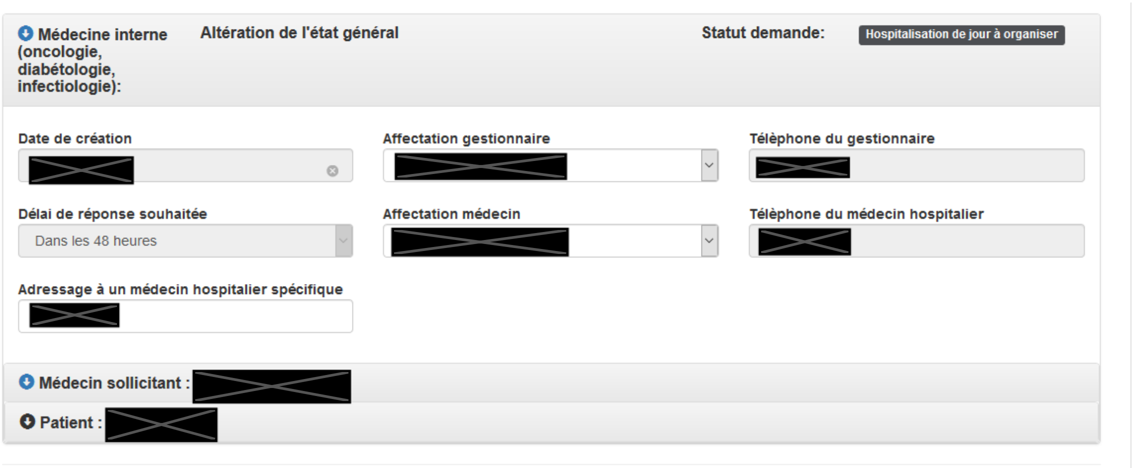

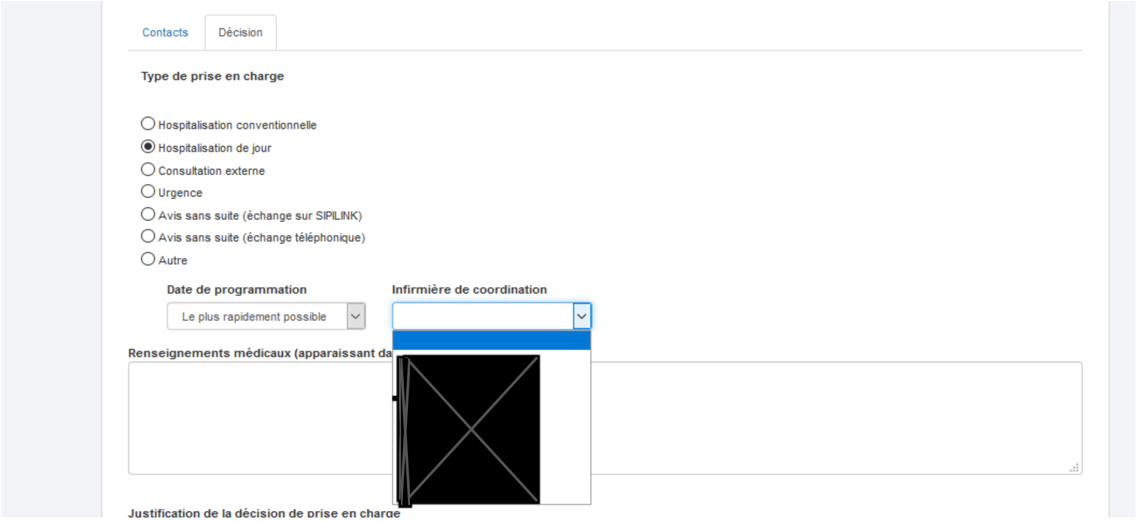
Contacts
Décision
Type de prise en charge
Hospitalisation conventionnelle
Hospitalisation de jour
Consultation externe
Urgence
Avis sans suite (échange sur SIPILINK)
Avis sans suite (échange téléphonique)
Autre
Date de programmation
Le plus rapidement possible
Infirmière de coordination
[redacted]
Renseignements médicaux (apparaissant da
Justification de la décision de prise en charge

1

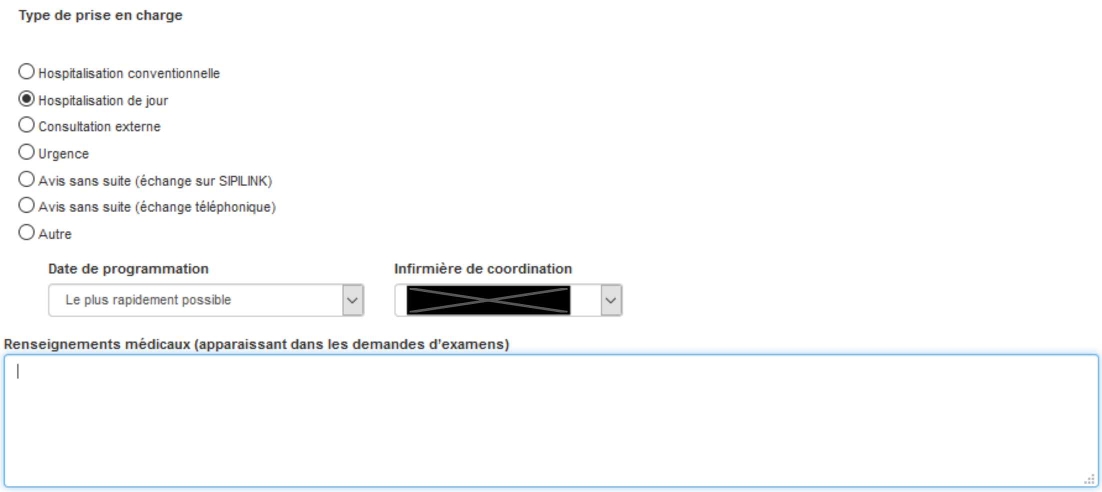
Type de prise en charge
Hospitalisation conventionnelle
Hospitalisation de jour
Consultation externe
Urgence
Avis sans suite (échange sur SIPILINK)
Avis sans suite (échange téléphonique)
Autre
Date de programmation
Le plus rapidement possible
Infirmière de coordination
[redacted]
Renseignements médicaux (apparaissant dans les demandes d'examens)

2

3
